# Toxic Metals Content in Impacted Third Molars and Adjacent Bone Tissue in Different Groups of Patients

**DOI:** 10.3390/ma14040793

**Published:** 2021-02-07

**Authors:** Ewelina Bryła, Maciej Dobrzyński, Damian Konkol, Piotr Kuropka, Marzena Styczyńska, Mariusz Korczyński

**Affiliations:** 1 Private Dental Clinic Maciej Kozlowski, Spokojna 23, 56-400 Olesnica, Poland; ewelinabryla@onet.pl; 2 Department of Pediatric Dentistry and Preclinical Dentistry, Wroclaw Medical University, Krakowska 26, 50-425 Wroclaw, Poland; 3 Department of Environment Hygiene and Animal Welfare, Wroclaw University of Environmental and Life Sciences, Chelmonskiego 38C, 51-630 Wroclaw, Poland; mariusz.korczynski@upwr.edu.pl; 4 Department of Histology and Embryology, Wroclaw University of Environmental and Life Sciences, Norwida 31, 50-375 Wroclaw, Poland; piotr.kuropka@upwr.edu.pl; 5 Department of Human Nutrition, Wroclaw University of Environmental and Life Sciences, Chelmonskiego 37/41, 51-630 Wroclaw, Poland; marzena.styczynska@upwr.edu.pl

**Keywords:** bioelements, teeth, molars, toxic metals

## Abstract

The aim of the study was to determine the content of: Pb, Cd, Cr, Ni, Fe, Mn, Cu, Zn in the impacted third molars and a fragment of covering bone. Patients were divided according to following criteria: place of residence, age, gender, anatomical location of the removed tooth. Content of Cd, Pb, and Mn in the samples increases with age of the patient. The content of Cd and Pb in the tooth and bone was higher in patients living in Wroclaw. Residents of the Wroclaw had higher Cu content in the teeth, while they had a lower content in the jawbone. In contrast to Cd and Pb, an average of 68% higher concentration of Zn in the jawbone than in the tooth was noted. The content of Cr in tooth was lower by 33% than in the mandible bone and, similarly to the content of Ni, it decreased with age. In women, the Fe content in bone of the jaw was higher than in the removed tooth. The presence of Pb and Cd aggregates is confirmed in all hard tissues of the tooth and bone, in contrast to bioelements which show a stronger tendency to aggregate, essentially within the dentine.

## 1. Introduction

Metals are elements commonly found in the environment. They form natural deposits occurring all over the world and are widely used in many areas of life. Some of them are necessary for life and the proper functioning of the organism, others do not play an important role in physiological processes, and in addition they can contribute to the defect of individual tissues and organs, impair their functions, and cause the whole spectrum of diseases in the mechanism of heavy poisoning resulting from intensive supply and chronic exposure to relatively low doses [1,2]. The toxic effect of metals consists primarily in blocking important functional groups in proteins, displacing ions that are cofactors for enzymes and other functional proteins and changing the spatial structure of proteins. This leads to a disruption of protein function in cells and in extreme cases can lead to death [3]. Due to the varying degree of toxicity, metals are classified into the following groups [4]:

- very high degree of potential danger: chromium (Cr), cadmium (Cd), copper (Cu), lead (Pb), mercury (Hg), zinc (Zn);
- high degree of potential danger: manganese (Mn), molybdenum (Mo), iron (Fe);
- average potential hazard: nickel (Ni), cobalt (Co);
- low potential hazard: zirconium (Zr), strontium (Sr).

Toxic metals and their derivatives can enter the human organism by inhalation, ingestion and as a result of skin absorption [5,6]. Although metal migration in the body is limited by the presence of biological barriers, however, with excessive supply of toxic elements, the effectiveness of these barriers is limited. The circulation of these metals within ecosystems is primarily associated with plant-animal-human food chains [6,7]. This is due to the fact that heavy metals are taken up by plants from the soil along with water and accumulate them in their tissues (mainly roots and leaves), which are eaten by animals and humans [8,9]. However, the amount of absorbed metal often depends on its form, pH, passage speed through the digestive system, and finally the presence of other substances that modify its absorption. Monitoring of these substances may be based on the use of bioidentifiers, which may be mineralized tissues, such as bones and teeth [10]. The main component of mineralized tissues is hydroxyapatite. Despite the fact that bones and teeth are chemically solid, it is believed that certain trace elements may accumulate in them by replacing calcium in the hydroxyapatite structure. Morphologically, there are two types of mature bone tissue: compact and spongy. Spongy bone can provide information on not too distant exposure to metals, while within compact bone, trace elements can accumulate for a much longer period. This is due to the different intensity of the reconstruction process. During the formation of mineralized tooth tissues, there is no release of mineral substances. In turn, in the case of bone tissue, in many physiological and pathological conditions, the release and fluctuation of mineral substances from the bones into the blood occurs. However, both deciduous teeth were lost as a result of physiological dentition replacement, as well as erupted permanent teeth that have been removed due to carious disease, periodontal disease, or lack of space in the dental arch have limited usefulness in biomonitoring. An important factor affecting the credibility of this type of test results is the fact that tooth enamel is constantly in direct contact with the oral environment, so various factors within the oral cavity, i.e. food, drugs or caries, can modify the mineral composition of the surface layers of the tooth. Research by Malara et al. [11] shows that a fully developed tooth that has completely retained in bone can be a valuable and objective substrate for the biomonitoring of toxic metals. Such a tooth is completely isolated from the oral environment, and the only source of minerals supplied during the process of creating hard substances of the tooth is blood. Third molars are most likely to retain, especially in the lower jaw. Wisdom teeth are fully developed and ready to erupt at the age of 17–25, while all other teeth are present in the dental arch of the mouth and bone growth is already completed. Surgical wisdom tooth extraction performed for surgical or orthodontic indications, after skillful removal of the bone tissue covering it, is a procedure very often performed in modern surgical dentistry. A removed tooth together with a directly covering fragment of bone tissue may constitute valuable material for scientific purposes. A feature of such a set of mineralized tissues (tooth-bone) is their origin in one anatomical area and complete histological differentiation. The peculiarity of the jawbone is the unusual morphology, as it is formed of a relatively thick layer of compact lamellar bone and a very small amount of spongy bone. Due to the fact that the retained lower wisdom teeth are almost exclusively covered by compacted bone, there is a possibility of long-term assessment of toxic metal accumulation. The aim of the study was to determine the content of selected toxic metals and microelements (Pb, Cd, Cr, Ni, Fe, Mn, Cu, Zn) in impacted third molars and in a fragment of bone tissue covering them, coming from the jaw of the different groups of patients-factors taken into account include place of residence, age, sex, anatomical location of the removed tooth, and histochemical detection of complexes of some toxic metal ions and elements within the mineralized tissues of the tooth.

## 2. Materials and Methods

Prior to the study, the consent of the Bioethics Committee of the Wroclaw Medical University was obtained (consent number: KB-61/2017).

### 2.1. Material

The material consisted of lower impacted third molars and a fragment of the bone tissue covering them obtained from 60 patients: 30 residents of the Wroclaw district (P) and 30 residents of the city of Wroclaw (M), who underwent surgery to extract completely retained teeth. Patients of both sexes were divided into the 3 age groups treated as young (18–27 years), mature (28–38 years), aged (39–65 years). Data on sex, age, permanent residence, no occupational exposure to toxic metals, and no smoking addiction and no use of dietary supplements were collected in the course of the subject study.

### 2.2. Morphological Studies

Morphological studies and their assessment were performed at the Department of Histology and Embryology at the Wroclaw University of Environmental and Life Sciences. They were intended to visualize the occurrence of metals in tooth tissues and adjacent periodontal tissues. For this purpose, a combined histochemical technique performed directly on the tooth and epifluorescence were used. For morphological studies, randomly selected teeth from two main research groups (P or M) from three age groups (18–27 years, 28–38 years, 39–65 years) were used, at least one tooth of both sexes. A total of 30 teeth were selected for morphological and histological studies.

The teeth and bone fragments were cut into 0.5 mm longitudinal or transverse sections with a water-cooled slow-speed diamond saw (EXAKT 310 CP) and then fixed in a 4% solution of buffered formalin. After rinsing in running water, the material was dyed to detect the presence of selected metals. To demonstrate calcium, Mallory staining was used, according to which calcium is stained red regardless of the test method. When analyzing the occurrence of iron, staining with the use of Prussian blue (blue aggregates) was used, while copper-rhodanine at acidic pH (red-brown-gold aggregates) was used. Ditizone was used to detect lead accumulation (black-brown fluorescence aggregates, red under transmitted light), which is a non-specific reaction. Due to the specificity of the analyzed material, minor Kuropka staining modifications were used.

The material after dyeing was dehydrated in an alcohol series and analyzed using a Nikon ECLIPSE 80i fluorescence microscope (Nicon Istruments Inc., Tallahassee, FL, USA) using a UV-2A and B-2A filter. The use of these two filters allowed the observation of reactive areas against the background of autofluorescent collagen fibers and other components of the tooth and periodontal tissues.

### 2.3. Determination of the Content: Pb, Cd, Cr, Ni, Fe, Mn, Cu, and Zn in Wisdom Teeth and Adjacent Bone Tissue

Determination of the content of selected mineral and toxic trace elements in bone tissue was performed in the Food Research Laboratory (PCA accreditation No. AB1396) of the Wroclaw University of Environmental and Life Sciences.

#### 2.3.1. Mineralization of Research Material

Mineralization of samples was carried out wet in a closed microwave system. To the sample weight of a homogeneous sample (from 0.1 g to 0.5 g), 5 cm^3^ of concentrated nitric acid (V) A.C.S. and 1 cm^3^ concentrated hydrogen peroxide A.C.S. was added, then the samples were mineralized in the MARS 5 microwave sample preparation system. The minerals were quantitatively transferred to 10 cm^3^ measuring vessels using redistilled water. Mineralization was carried out in accordance with the Polish Standard PN-EN 13805: 2003 Food products—Determination of trace elements—Pressure mineralization [12].

#### 2.3.2. Determination of Elements in the Research Material

Determination of Pb, Cd, Cr, Ni, Fe, Mn, Cu, Zn content was performed by atomic absorption spectrometry in an air-acetylene flame using the SpectraAA atomic absorption spectrometer with a V2 AA240FS flame attachment, using dedicated hollow cathode lamps. The accuracy of the method was confirmed on the basis of the certified reference material ERM-BD151 Skimmed milk powder (Sigma-Aldrich, Saint Louis, MO., USA), and the measurement uncertainty was estimated at 5%. The elements were determined according to the following standards:

- Pb, Cd, Zn, Cu, Fe, Cr—PN-EN 14082: 2004 Food products—Determination of trace elements—Determination of lead, cadmium, zinc, copper, iron, and chromium by atomic absorption spectrometry (AAS) after dry mineralization [13].
- Ni—PN-A-86939-6: 1998 Vegetable and animal oils and fats—Determination of heavy metal content by atomic emission spectrometry—Determination of nickel content [14].
- Mn—PN-EN ISO 6869: 2002 Feed—Determination of calcium, copper, iron, magnesium, manganese, potassium, sodium, and zinc content—Atomic absorption spectrometry method [15].

Table 1 presents the parameters of the measurements taken.

### 2.4. Statistical Analysis of the Results

Statistical analyzes were performed using Statistica ver. 12 and an Excel spreadsheet. The mean value and standard deviation (SD) were calculated for each quantitative variable. The compliance of the empirical quantitative variable distributions with the theoretical normal distribution was verified using the Shapiro-Wilk test. To assess the significance of differences between the average values of quantitative parameters in two independent groups (place of residence, gender) in the case of variables with normal distribution, Student’s *t*-test for independent variables was used, or the non-parametric Mann-Whitney U test when the distribution of a variable in one of the groups it differed significantly from the normal distribution. The significance of differences between the average values of quantitative parameters in two related groups (place of sampling) was verified on the basis of Student’s *t*-test for dependent variables or Wilcoxon test. For a larger number of groups (age groups), one-way analysis of variance or its non-parametric equivalent—the Kruskal-Wallis test—was used.

## 3. Results

### 3.1. Characteristics of Patients of Both Groups

Table 2 contains basic statistics of the characteristics of patients from both groups (M and P). Both groups of patients did not differ significantly in terms of sex structure, age, and location of the removed tooth (*P* > 0.05).

### 3.2. The Content of Toxic Metals and Bioelements in Wisdom Teeth and Jawbone of Patients from Both Groups (P and M)

Basic statistics of toxic metal content in tested samples from patients from both groups are presented in Table 3, Table 4, Table 5, Table 6, Table 7, Table 8, Table 9, Table 10, Table 11, Table 12, Table 13, Table 14, Table 15, Table 16, Table 17, Table 18, Table 19, Table 20, Table 21, Table 22, Table 23, Table 24, Table 25 and Table 26.

#### 3.2.1. Content of Cadmium

The results of measurement of cadmium content in the removed tooth (tooth) and mandibular bone tissue (bone) in subgroups of patients differing in place of residence, sex, and age as well as the results of comparisons are presented in Table 3, Table 4 and Table 5.

Cadmium contents in both the tooth and bone tissue were statistically significantly higher in patients living in Wroclaw (*P* < 0.05).

No statistically significant effect of patients’ sex on tooth and bone cadmium content was observed (*P* > 0.05).

The cadmium content in the tested samples increases with the age of patients. In removed teeth, the differences are statistically significant (*P* < 0.001). In all age groups, the cadmium content in the tooth was lower than in bone tissue, however the differences were not statistically significant (*P* > 0.05).

#### 3.2.2. Content of Lead

The results of the measurement of lead content in the removed tooth (tooth) and mandibular bone tissue (bone) in patient subgroups differing in place of residence, sex, and age as well as the results of comparisons are presented in Table 6, Table 7 and Table 8.

The lead content in the teeth and bone tissue of the jaw was significantly lower in patients living in the Wroclaw district (*P* < 0.05). In patients living in Wroclaw, the lead content in bone tissue was higher than in the tooth (*P* < 0.05).

The lead content in the teeth and bone tissue of the jaw was significantly lower in patients living in the Wroclaw district (*P* < 0.05). In patients living in Wroclaw, the lead content in bone tissue was higher than in the tooth (*P* < 0.05).

No statistically significant effect of patient sex on lead content in tooth and bone was observed (*P* > 0.05). In women, the lead content in the bone tissue of the jaw is higher than in the removed teeth (*P* < 0.05).

The lead content in the tested samples increases with the age of patients. In removed teeth, the differences are statistically significant at the level of *P* < 0.001, and in bone tissue at the level of *P* = 0.001. In all age groups, the lead content in the tooth was lower than in bone tissue, but these differences were not statistically significant (*P* > 0.05).

#### 3.2.3. Content of Zinc

The results of measuring the zinc content in the removed tooth (tooth) and mandibular bone tissue (bone) in subgroups of patients differing in place of residence, sex, and age as well as the results of comparisons are presented in Table 9, Table 10 and Table 11.

No statistically significant difference was observed between the average zinc content in the tooth and jawbone and the place of residence (*P* > 0.05). In the group of residents of the Wroclaw district, the zinc content in the bone tissue of the jaw was higher than in the removed tooth (*P* = 0.006).

No statistically significant effect of patients’ sex on the content of zinc in the tooth and bone was observed (*P* > 0.05). In men, the concentration of zinc in the bone tissue of the jaw is higher than in the removed teeth (*P* < 0.05).

The zinc content in the tested samples does not significantly depend on the age of the patients (*P* > 0.05). In the 39–65 age group, the zinc content in the tooth was significantly lower than in bone tissue (*P* < 0.01).

#### 3.2.4. Content of Nickel

The results of measuring the content of nickel in the removed tooth (tooth) and mandibular bone (bone) in subgroups of patients differing in place of residence, sex, and age, and the results of comparisons are presented in Table 12, Table 13 and Table 14.

The nickel content did not depend on the patients’ place of residence or the type of sample (*P* > 0.05).

No statistically significant effect of patient sex on nickel content in tooth and bone was observed (*P* > 0.05). 

In the 39–65 age group, the nickel content in the tooth was significantly lower than in the other age groups (*P* < 0.05). In addition, the content of this element was significantly higher in bone than in the teeth of patients in the 18–27 age group (*P* < 0.05).

#### 3.2.5. Content of Chromium

The results of the measurement of chromium content in the removed tooth (tooth) and jawbone tissue (bone) in subgroups of patients differing in place of residence, sex, and age, and the results of comparisons are presented in Table 15, Table 16 and Table 17.

The content of chromium in the bone tissue of the jaw of patients living in the Wroclaw district was significantly higher than in patients from Wroclaw (*P* = 0.001). In patients from the Wroclaw district, the content of chromium in teeth was lower than in bone tissue (*P* < 0.01).

No statistically significant effect of patients’ sex on the content of chromium in tooth and bone (*P* > 0.05) was observed.

The content of chromium in a tooth decreases with the age of patients, while in the teeth of people aged 18–27 it was significantly higher than in other age groups (*P* < 0.001). In the 39–65 age group, the content of chromium in the removed tooth was lower than in bone tissue (*P* < 0.05).

#### 3.2.6. Content of Iron

The results of measurement of iron content in the removed tooth (tooth) and mandibular bone tissue (bone) in subgroups of patients differing in place of residence, sex, and age as well as the results of comparisons are presented in Table 18, Table 19, Table 20 and Table 21.

The iron content did not depend on the patients’ place of residence (*P* > 0.05), but it was higher in the bone tissue of the jaw than in the removed tooth. In the group of patients living in the Wroclaw district, the difference was statistically significant at the level of *P* < 0.01.

No statistically significant effect of patients’ sex on iron and bone content was observed (*P* > 0.05). In women, the iron content in the bone tissue of the jaw was higher than in the removed tooth (*P* < 0.05).

The iron content of a tooth has no statistically significant relationship with the age of patients. The content of this element in the teeth of patients in all three age groups was lower than in bone tissue, but these differences were not statistically significant (*P* > 0.05).

#### 3.2.7. Content of Copper

The results of measurement of copper content in the removed tooth (tooth) and mandibular bone tissue (bone) in subgroups of patients differing in place of residence, sex, and age, and the results of comparisons are presented in Table 21, Table 22 and Table 23.

Statistically significant differences between the average level of copper were observed only in groups differing in the place of residence. In group M (residents of Wroclaw) the copper content in the mandible bone was lower (*P* < 0.001) than in group P (Wroclaw district). Significantly higher copper content in teeth (*P* = 0.001) was observed in the inhabitants of Wroclaw, while there was lower content in jawbone tissues (*P* = 0.035).

#### 3.2.8. Content of Manganese

The results of measuring the content of manganese in the removed tooth (tooth) and mandibular bone (bone) in patient subgroups differing in place of residence, sex, and age, and the results of comparisons are presented in Table 24, Table 25 and Table 26.

The content of manganese in the teeth was dependent on the patients’ place of residence (*P* < 0.05). It was also significantly smaller in the bones of patients from both experimental groups (*P* < 0.05).

There was no significant effect of the patient’s sex on the manganese content in the samples (*P* > 0.05), while the content of this element in the removed teeth was significantly higher than in the bone tissue of the jaw (*P* < 0.01).

The content of manganese in the examined samples increases with the age of patients, but only in the removed teeth of patients aged 39–65 is significantly higher than in patients aged 18–27 (*P* < 0.001). In all age groups, the content of manganese in the teeth was higher than in the bone tissue of the jaw, but only in the 28-38 age group was this difference statistically significant (*P* < 0.001).

### 3.3. Histochemistry

In order to determine the content of selected elements in the tested material, the chemical (combustion) method was used, while the histochemical reactions were aimed at locating the sites of the main deposition of metal aggregates. Illustratively, calcium—the basic structural bio-element of mineralized tissues, and toxic metals—lead and cadmium, as well as copper.

#### 3.3.1. Analysis of the Presence of Calcium

The calcium distribution in the analyzed material turned out to be different and individual-specific. It most often formed accumulations in the form of balls surrounding dentinal tubules. In some cases, extensive non-responsive areas were visible in the dentine. They were probably zones with reduced mineralization, which could have been caused by the presence of other elements, e.g. copper or lead. Such a picture was observed in all examined groups.

Within the enamel, calcium was evenly distributed in young people (from the first age group). On the other hand, within the tooth crown in people aged 39–65, where the phenomenon of aging was clearly marked, the enamel was cracked and contained areas of leached calcium. For bones and cement, calcium was distributed correctly and showed no disturbance.

#### 3.3.2. Analysis of the Presence of Iron

Iron in dentine was in the form of small aggregates spaced at regular intervals from each other. For women, sometimes they formed clear boundary lines with increased iron content. The character of aggregates in dentin did not vary depending on age and was similar in all age groups. There was no positive iron reaction in the enamel, except in two cases where a positive reaction was found on the surface of this tissue. Its presence can be associated with the residue of hemolyzed blood that remained on the tooth after extraction. Within cement and bone tissue, a positive reaction was observed in a few areas—within the matrix—and in adjacent tissues.

#### 3.3.3. Analysis of the Presence of Lead, Cadmium, and Copper

Positive reactions with toxic metals (lead, cadmium) were observed in all hard tissues of the tooth and in adjacent bone tissue. Toxic metal aggregates were found mainly in dentin adjacent to the dental canal or in the central part of dentin. In addition, particularly numerous aggregates of aggregates occurred within the bone tissue. Aggregates were most rarely observed in the enamel area. Aggregates in dentin were most often arranged along the dentinal tubules with numerous gaps in relation to the enamel–tooth cavity direction. The most numerous aggregates were found in the 39–65 age group both in the enamel area and in the entire volume of dentin. On the other hand, in relation to copper, in a few cases it was found in enamel, although its strongest reaction was obtained in dentin adjacent to the tooth cavity.

## 4. Discussion

In the own material studied, for most elements, significant differences were found between their content in teeth and bone, especially in lead, which can indicate an improvement in the quality of the environment in relation to toxic metals. However, it should be emphasized that not only toxic metals, but also various types of organic derivatives, including dioxins, are responsible for environmental pollution [16]. The conducted research focused on bioelements such as zinc, iron, manganese, copper, whose content affects the functioning of hard tissues. In addition, the basic structural element of mineralized tissues and calcium was visualized in morphological studies.

The study found that the concentration of this element did not depend on the place of residence, however, women found a higher concentration of iron in bone tissue than in teeth. This fact can be explained by the fact that iron is one of the trace elements present in bones, which accumulates with age, and its content depends mainly on the level in the blood serum. In states of excess of this element, liver protein, heptidine, inhibits the absorption of iron from erythrocytes into the circulation, and also reduces the release of iron from macrophages [17]. Similar observations were made by Maciejewska [18], who also paid special attention to the impact of other trace elements on bone development.

Maciejewska’s research on the concentration of zinc in young rats showed a decrease in the concentration of zinc in bones with age and a slightly higher concentration of this element in incisal teeth than in bones. The obtained results of our own research indicate that in most cases the concentration of zinc does not depend on the patient’s age or de facto place of residence. The above element showed a very diverse content in the examined material, generally higher in bones than in teeth. It is very likely that such different values can be associated with different rat and human life expectancy, as well as different bone structure [16]. In two patients from Wroclaw, an excessive content of zinc in the bone was found, which was not found in the tooth. Such a high concentration can be formed only as a result of long-term zinc supplementation or as a result of a diet based on some plant foods rich in this element, which include, among others sunflower, pumpkin, wheat germ, and bulbous seeds [19,20].

Wychowański and Małkiewicz [2] analyzed the occurrence of trace elements in the enamel and dentine of third retained molars in patients staying in the urban and agricultural areas of Mazovia. These authors took dental material from 30 patients aged 26-37. The results of their research indicate that the enamel and dentine in people living in the city contain a significantly higher level of lead and cadmium than those living in agricultural areas, as well as the level of manganese and chromium. In the own research, in the case of city residents, an increased content of cadmium in tooth tissues was also noted, which increased with age, but was independent of gender.

In the case of chromium, its content was higher in the inhabitants of the Wroclaw district than in the cities of Wroclaw, while the level of this element was lower in the teeth than in the bone tissue. In addition, a significantly higher content was confirmed in young patients compared to older ones. This element is used as a component of preparations for reducing body weight [21]. Long-term elevated levels of chromium, which accumulated in the bone samples and teeth tested, can only be explained by the use of slimming preparations or by prolonged increase of its content in drinking water. 

In studies from the Mazovia region carried out by Wychowański and Małkiewicz [2], no differences in the content of manganese in tooth tissues between residents of urban and agricultural areas were found. In this research it was found that the concentration of this element in the teeth was statistically higher than in bone samples and increased with age in patients in the 39–65 age range. Deficiency or excess of manganese can be associated with malabsorption or excessive pharmacological supplementation. Since healthy people without neuropathological changes or skeletal structure were qualified for the study, it should be assumed that the manganese concentration was normal and the observed increase in its content in hard tissues should be assessed as one of the natural physiological buffer phenomena. Such a process is bone remodeling and a gradual increase in the level of dentin and root cement mineralization in the course of individual life. These are mechanisms that allow the accumulation of excess manganese present in the blood in mineralized tissues [22]. This protects the cells of other tissues from its excess or deficiency. 

In the conducted own research, the inhabitants of Wroclaw showed significantly lower copper concentration in the bone than the inhabitants of the district. This can be explained by the old water supply infrastructure in the district, the more frequent use of copper vessels, and in some cases living in the area of ironworks Siechnice. In turn, the inhabitants of Wroclaw had higher levels of copper in their teeth than in bones. Bone tissue undergoes constant remodeling processes, which may lead to a decrease in copper content in this tissue in the long term. Due to the fact that the hard tissues of the tooth are not remodeled, they maintained an increased copper content in accordance with the time when they were formed. No changes in copper content occurred with age, which contradicts the study by [23], which assessed the content of elements in permanent teeth in children aged 5–14 years. They found an increasing concentration of copper and other elements in older children. This contradiction may result from the fact that the age of the examined individuals did not exceed 14 years old and concerned children undergoing constant development, relatively briefly exposed to the environment. It seems that after full saturation of the tooth’s hard tissues with minerals, which can last for a few years after the tooth’s root, it remains constant and no longer increases during life. In our own research, there was no correlation between copper content in teeth and age in people aged 19–65.

Wychowański and Małkiewicz [2] found lower levels of lead in teeth in people from rural areas than in urban agglomeration. However, they did not take more into account the change in lead content tendencies depending on age and gender. Similarly, the inhabitants of Wroclaw had a higher lead content compared to the inhabitants of the district, however it was higher in bone tissue than in teeth. In women, the lead content in bone tissue was higher than in the tooth, and in both sexes it increased with age. Such oversupply of lead in mineralized tissues can only be associated with the environmental incorporation of this element. These results have been confirmed by many researchers [24,25,26].

Slow accumulation of nickel in hard tissues progresses with age, which was confirmed in our own research. Nickel is rarely the subject of tissue elemental analysis [27]. According to this author, an excess of nickel can negatively affect the enamel formation process, mainly in terms of hydroxyapatite crystal size. In the analyzed samples it was found that in the 18–27 age group the nickel content in the tooth was significantly lower than in the other age groups. Since no differences were found depending on gender and place of residence, it can be concluded that the nickel content in the surroundings decreased both in the city and in the district. One of the mechanisms that can lead to increased nickel content is orthodontic treatment with fixed braces [28]. Considering the fact that our own research was carried out on completely retained teeth, the possibility of nickel penetration into tooth tissues and adjacent tissues from permanent orthodontic appliances (in cases of their use) is excluded, which is also confirmed by experimental studies on pigs [29].

Combined morphological studies and chemical analysis allowed for accurate attribution of the presence of metals to the appropriate hard tissues of the tooth.

The most important advantage of the conducted research was the fact that by chemical analysis of completely retained teeth, the possibility of metallic contamination of their surface with material coming directly from the oral cavity or iatrogenic (orthodontic appliances) was excluded [29]. In numerous research works carried out in Poland and abroad, teeth that were already erupted, diseased (caries), or from fossil material were used. By avoiding biological or chemical contamination by choosing a completely retained tooth model and including morphological analysis in the scope of research, it was obtained certainty that the examined elements were tooth content, and did not come from adjacent tissues or from the oral cavity. Some authors conducted research on primary teeth as easy to obtain in the course of the physiological process of dentition replacement. However, the mineral composition assessed was rather a reflection of the mother’s intoxication state than a reflection of the state of the environment. In addition, the teeth do not mineralize in a short time, but it is a long-term process. During this period, newborns and children up to 10 years of age can be considered isolated from the environment, so the teeth remain with traces of the dietary regime imposed by parents and the social conditions in which they are brought up. An example would be lead toys, which were popular in the past and which, depending on availability in a given family, could have exposed the child to lead incorporation. Iron and copper occur in the dentine, cement and alveolar bone, forming specific aggregates, which confirms their participation in collagen synthesis. There were no aggregates on the tooth surface or enamel.

The aim of the study was to determine the content of selected toxic metals and microelements in impacted third molars and in a fragment of bone tissue covering them. However, it should be remembered that tooth extraction can cause bone tissue cavities. Filling cavities after tooth extraction should constitute future research directions. The use of stem cells may be effective in this aspect. The ability to differentiate stem cells into specific cell lines with the possibility of almost unlimited self-renewal and release of trophic/immunomodulatory factors may be an attractive source for bone and dental regeneration. However, to develop novel regenerative tools in stem cell dentistry, research is needed to further discover the plasticity of stem cells to identify their optimal sources and features [30]. Interesting prospects are also provided by the use of biomaterials such as magnesium-enriched hydroxyapatite and corticocancellous porcine bone. The analysis of the research results showed the absence of inflammatory cells and bone formation in all treated areas. In addition, the bones treated in this way were characterized by greater vitality [31,32].

Based on the obtained results, it can be concluded that the content of cadmium, lead and manganese in the tested samples increases with the age of the patient, mainly in tooth tissues, which may indicate that older patients were exposed to higher concentrations of these elements in the air during tooth development, which show greater cumulative capacity than bone tissue. The content of cadmium and lead in both the tooth and bone tissue was statistically significantly higher in patients living in Wroclaw, which may indicate greater air pollution in the city during the mineralization process. Residents of the Wroclaw agglomeration had significantly higher copper content in the teeth (*P* = 0.001), while a lower content in the jawbone (*P* = 0.035). In contrast to cadmium and lead, an average of 68% higher concentration of zinc in the jawbone than in the tooth tissues was noted. The content of chromium in tooth tissues was lower by 33% than in the mandible bone and, similarly to the content of nickel, it decreased with age. In women, the iron content in the bone tissue of the jaw was higher than in the removed tooth (*P* < 0.05), which may be associated with increased retention of this element, compensating for its losses during the menstrual phase of the monthly cycle. Histochemical methods are useful in the simple detection of possible metal aggregation sites in mineralized tissues and are a valuable complement to quantitative studies. Calcium as the basic component of teeth and bones aggregates in the form of balls surrounding dentinal tubules regardless of age and sex. In turn, iron and copper show the ability to accumulate in the form of small, regularly distributed aggregates, occurring practically only within the dentine. The presence of lead and cadmium aggregates is confirmed in all hard tissues of the tooth and in adjacent bone tissue, in contrast to bioelements (Ca, Cu, Fe), which show a strong tendency to aggregate essentially within the dentine.

## Figures and Tables

**Table 1:** Measurement parameters.

Determined Element	Wavelength (nm)	Gap (nm)	Air-Acetylene Flow (L/min)	Background Correlation	Characteristic Concentration c (mg/L)	r
Pb	217.0	1.0	13.50/2.00	on	0.005	0.9997
Cd	228.8	0.5	13.50/2.00	on	0.005	0.9998
Cr	425.4	0.2	13.50/2.00	on	0.005	0.9993
Ni	341.5	0.2	13.50/2.00	on	0.005	0.9997
Fe	372.0	0.2	13.50/2.00	on	0.011	1.0000
Mn	279.5	0.2	13.50/2.00	on	0.009	0.9996
Cu	327.4	0.2	13.50/2.00	on	0.015	0.9999
Zn	213.9	1.0	13.50/2.00	on	0.012	1.0000

r—Pearson’s correlation coefficient.

**Table 2:** Characteristics of patients.

Feature	M (N = 30)		*P* (N = 30)		All		*p*-Value
	n	%	n	%	n	%	
Sex							
Women	17	56.7	16	53.3	33	55	1.00
Men	13	43.3	14	46.7	27	45	
Tooth location							
38	21	70.0	16	53.3	37	61.7	0.288
48	9	30.0	14	46.7	23	38.3	
Age group							
18–27	6	20.0	11	36.7	17	28.3	0.266
28–38	15	50.0	14	46.7	29	48.3	
39–65	9	30.0	5	16.6	14	23.4	
Age							
Mean ± SD	35.2 ± 10.0		31.8 ± 9.2		33.5 ± 9.7		0.143

**Table 3:** Cadmium content in tested samples taken from patients differing in place of residence.

Cadmium Content (μg/g)	M (N = 30)	*P* (N = 30)	*p*-Value
Tooth (mean ± SD)	1.322 ^a^ ± 0.629	0.898 ^b^ ± 0.693	0.017
Bone (mean ± SD)	1.593 ^a^ ± 0.937	0.860 ^b^ ± 0.906	0.003
*p*-value	0.101	0.567	-

^a, b^—statistically significant differences at the level of *P* < 0.05.

**Table 4:** Cadmium content in tested samples taken from patients of different sexes.

Cadmium Content (μg/g)	Women (N = 33)	Men (N = 27)	*p*-Value
Tooth (mean ± SD)	1.063 ± 0.629	1.167 ± 0.693	0.338
Bone (mean ± SD)	1.288 ± 0.629	1.152 ± 0.693	0.508
*p*-value	0.121	0.696	-

**Table 5:** Cadmium content in tested samples taken from patients of different age.

Cadmium Content (μg/g)	18–27 (N = 17)	28–38 (N = 29)	39–65 (N = 14)	*p*-Value
Tooth (mean ± SD)	0.632 ^a^ ± 0.385	1.053 ^b^ ± 0.632	1.809 ^c^ ± 0.537	<0.001
Bone (mean ± SD)	0.929 ± 0.916	1.104 ± 0.777	1.841 ± 1.233	0.058
*p*-value	0.163	0.810	0.875	-

^a, b, c^—statistically significant differences at the level of *P* < 0.05.

**Table 6:** Lead content in tested samples taken from patients differing in place of residence.

Lead Content (μg/g)	M (N = 30)	*P* (N = 30)	*p*-Value
Tooth (mean ± SD)	10.142 ^ac^ ± 5.401	5.161 ^b^ ± 5.563	0.002
Bone (mean ± SD)	13.462 ^ad^ ± 8.818	6.106 ^b^ ± 8.090	0.002
*p*-value	0.029	0.600	-

^a, b, c, d^—statistically significant differences at the level of *P* < 0.05.

**Table 7:** Lead content in tested samples taken from patients of different sexes.

Lead Content (μg/g)	Women (N = 33)	Men (N = 27)	*p*-Value
Tooth (mean ± SD)	6.854 ^a^ ± 5.877	8.626 ± 6.084	0.268
Bone (mean ± SD)	10.241 ^b^ ± 8.102	9.226 ± 10.465	0.279
*p*-value	0.012	0.732	-

^a, b^—statistically significant differences at the level of *P* < 0.05.

**Table 8:** Lead content in tested samples taken from patients of different age.

Lead Content (μg/g)	18–27 (N = 17)	28–38 (N = 29)	39–65 (N = 14)	*p*-Value
Tooth (mean ± SD)	3.354 ^a^ ± 3.452	7.430 ^b^ ± 5.371	13.328 ^c^ ± 5.199	<0.001
Bone (mean ± SD)	5.764 ^a^ ± 6.988	7.790 ^b^ ± 6.544	18.797 ^c^ ± 10.590	0.001
*p*-value	0.078	0.597	0.140	-

^a, b, c^—statistically significant differences at the level of *P* < 0.05.

**Table 9:** Zinc content in tested samples taken from patients differing in place of residence.

Zinc Content (μg/g)	M (N = 30)	*P* (N = 30)	*p*-Value
Tooth (mean ± SD)	122.2 ± 198.3	78.3 ^a^ ± 34.0	0.363
Bone (mean ± SD)	471.2 ± 1376.3	159.2 ^b^ ± 172.5	0.352
*p*-value	0.222	0.006	-

^a, b^—statistically significant differences at the level of *P* < 0.05.

**Table 10:** Zinc content in tested samples taken from patients of different sexes.

Zinc Content (μg/g)	Women (N = 33)	Men (N = 27)	*p*-Value
Tooth (mean ± SD)	113.3 ± 185.6	83.0 ^a^ ± 44.0	0.772
Bone (mean ± SD)	299.1 ± 963.0	334.9 ^b^ ± 1029.5	0.683
*p*-value	0.102	0.013	-

^a, b^—statistically significant differences at the level of *P* < 0.05.

**Table 11:** Zinc content in tested samples taken from patients of different age.

Zinc Content (μg/g)	18–27 (N = 17)	28–38 (N = 29)	39–65 (N = 14)	*p*-Value
Tooth (mean ± SD)	98.1 ± 59.9	114.3 ± 200.7	73.3 ^a^ ± 15.2	0.850
Bone (mean ± SD)	167.6 ± 211.2	132.4 ± 136.3	873.2 ^b^ ± 1967.4	0.592
*p*-value	0.356	0.065	0.004	-

^a, b^—statistically significant differences at the level of *P* < 0.05.

**Table 12:** Nickel content in tested samples taken from patients differing in place of residence.

Nickel Content (μg/g)	M (N = 30)	*P* (N = 30)	*p*-Value
Tooth (mean ± SD)	1.309 ± 5.107	0.394 ± 0.790	0.363
Bone (mean ± SD)	1.246 ± 3.385	1.242 ± 2.421	0.352
*p*-value	0.717	0.159	-

**Table 13:** Nickel content in tested samples taken from patients of different sexes.

Nickel Content (μg/g)	Women (N = 33)	Men (N = 27)	*p*-Value
Tooth (mean ± SD)	0.425 ± 1.074	1.372 ± 5.324	0.758
Bone (mean ± SD)	1.231 ± 3.226	1.260 ± 2.552	0.663
*p*-value	0.159	0.642	-

**Table 14:** Nickel content in tested samples taken from patients of different age.

Nickel Content (μg/g)	18–27 (N = 17)	28–38 (N = 29)	39–65 (N = 14)	*p*-Value
Tooth (mean ± SD)	0.668 ^a^ ± 0.861	1.315 ^a^ ± 5.207	0.115 ^b^ ± 0.354	0.035
Bone (mean ± SD)	1.573 ± 2.905	0.843 ± 1.561	1.675 ± 4.723	0.526
*p*-value	0.038	0.547	0.069	-

^a, b^—statistically significant differences at the level of *P* < 0.05.

**Table 15:** Chromium content in tested samples taken from patients differing in place of residence.

Chromium Content (μg/g)	M (N = 30)	*P* (N = 30)	*p*-Value
Tooth (mean ± SD)	1.407 ± 2.643	1.613 ^a^ ± 2.669	0.252
Bone (mean ± SD)	1.083 ^a^ ± 2.560	3.450 ^b^ ± 4.576	0.001
*p*-value	0.677	0.002	-

^a, b^—statistically significant differences at the level of *P* < 0.05.

**Table 16:** Chromium content in tested samples taken from patients of different sexes.

Chromium Content (μg/g)	Women (N = 33)	Men (N = 27)	*p*-Value
Tooth (mean ± SD)	1.320 ± 2.081	1.743 ± 3.214	0.677
Bone (mean ± SD)	2.410 ± 4.369	2.092 ± 3.216	0.838
*p*-value	0.196	0.116	-

**Table 17:** Chromium content in tested samples taken from patients of different age.

Chromium Content (μg/g)	18–27 (N = 17)	28–38 (N = 29)	39–65 (N = 14)	*p*-Value
Tooth (mean ± SD)	2.793 ^a^ ± 3.434	1.418 ^b^ ± 2.431	0.143 ^c^ ± 0.306	<0.001
Bone (mean ± SD)	2.765 ± 3.815	2.243 ± 4.088	1.712 ^d^ ± 3.636	0.458
*p*-value	0.653	0.212	0.021	-

^a, b, c, d^—statistically significant differences at the level of *P* < 0.05.

**Table 18:** Iron content in tested samples taken from patients differing in place of residence.

Iron Content (μg/g)	M (N = 30)	*P* (N = 30)	*p*-Value
Tooth (mean ± SD)	5.312 ± 3.010	4.002 ^a^ ± 2.319	0.071
Bone (mean ± SD)	8.564 ± 13.221	6.317 ^b^ ± 3.463	0.701
*p*-value	0.131	0.007	-

^a, b^—statistically significant differences at the level of *P* < 0.05.

**Table 19:** Iron content in tested samples taken from patients of different sexes.

Iron Content (μg/g)	Women (N = 33)	Men (N = 27)	*p*-Value
Tooth (mean ± SD)	4.457 ^a^ ± 2.867	4.901 ± 2.620	0.302
Bone (mean ± SD)	8.628 ^b^ ± 12.725	5.988 ± 2.827	0.537
*p*-value	0.013	0.107	-

^a, b^—statistically significant differences at the level of *P* < 0.05.

**Table 20:** Iron content in tested samples taken from patients of different age.

Iron Content (μg/g)	18–27 (N = 17)	28–38 (N = 29	39–65 (N = 14)	*p*-Value
Tooth (mean ± SD)	4.943 ± 3.209	4.150 ± 2.472	5.360 ± 2.670	0.283
Bone (mean ± SD)	6.909 ± 4.035	5.417 ± 2.128	12.276 ± 18.922	0.074
*p*-value	0.136	0.058	0.084	-

**Table 21:** Copper content in tested samples taken from patients differing in place of residence.

Copper Content (μg/g)	M (N = 30)	*P* (N = 30)	*p*-Value
Tooth (mean ± SD)	0.895 ± 1.299	0.358 ± 0.901	0.184
Bone (mean ± SD)	0.026 ^a^ ± 0.099	0.928 ^b^ ± 1.718	<0.001
*p*-value	0.001	0.035	-

^a, b^—statistically significant differences at the level of *P* < 0.05.

**Table 22:** Copper content in tested samples taken from patients of different sexes.

Copper Content (μg/g)	Women (N = 33)	Men (N = 27)	*p*-Value
Tooth (mean ± SD)	0.608 ± 0.890	0.650 ± 1.406	0.184
Bone (mean ± SD)	0.485 ± 0.954	0.466 ± 1.628	0.225
*p*-value	0.465	0.594	-

**Table 23:** Copper content in tested samples taken from patients of different age.

Copper Content (μg/g)	18–27 (N = 17)	28–38 (N = 29)	39–65 (N = 14)	*p*-Value
Tooth (mean ± SD)	0.902 ± 1.379	0.519 ± 0.825	0.516 ± 1.399	0.117
Bone (mean ± SD)	0.506 ± 0.708	0.645 ± 1.748	0.094 ± 0.347	0.118
*p*-value	0.583	0.557	0.285	-

**Table 24:** Manganese content in tested samples taken from patients differing in place of residence.

Manganese Content (μg/g)	M (N = 30)	*P* (N = 30)	*p*-Value
Tooth (mean ± SD)	2.310 ^a^ ± 0.582	1.862 ^b^ ± 0.615	0.005
Bone (mean ± SD)	1.704 ± 0.847	1.520 ± 0.524	0.315
*p*-value	0.001	0.008	-

^a, b^—statistically significant differences at the level of *P* < 0.05.

**Table 25:** Manganese content in tested samples taken from patients of different sexes.

Manganese Content (μg/g)	Women (N = 33)	Men (N = 27)	*p*-Value
Tooth (mean ± SD)	2.162 ^a^ ± 0.620	1.992 ^a^ ± 0.653	0.306
Bone (mean ± SD)	1.767 ^b^ ± 0.626	1.422 ^b^ ± 0.758	0.059
*p*-value	0.006	0.001	-

^a, b^—statistically significant differences at the level of *P* < 0.05.

**Table 26:** Manganese content in tested samples taken from patients of different age.

Manganese Content (μg/g)	18–27 (N = 17)	28–38 (N = 29)	39–65 (N = 14)	*p*-Value
Tooth (mean ± SD)	1.707 ^a^ ± 0.681.	2.097 ^ab^ ± 0.521	2.524 ^b^ ± 0.531	0.001
Bone (mean ± SD)	1.497 ± 0.591	1.516 ^c^ ± 0.554	1.949 ± 1.000	0.122
*p*-value	0.195	<0.001	0.056	-

^a, b, c^—statistically significant differences at the level of *P* < 0.05.

## Data Availability

The data presented in this study are available on request from the corresponding author.
